# Emotional responses and engagement with misinformation in adolescents

**DOI:** 10.3389/fpsyg.2026.1858557

**Published:** 2026-07-14

**Authors:** Ecaterina Ilis, Razvan Ilis, Katarzyna Molek-Kozakowska, Simina-Maria Terian

**Affiliations:** 1 Faculty of Letters and Arts, Lucian Blaga University of Sibiu, Sibiu, Romania; 2 Faculty of Social and Human Sciences, Lucian Blaga University of Sibiu, Sibiu, Romania; 3 Institute of Linguistics, University of Opole, Opole, Poland

**Keywords:** adolescents, credibility, emotion, misinformation, news literacy, Russia–Ukraine war, social media

## Abstract

Adolescents are increasingly exposed to misinformation in social media environments where emotionally salient content competes with verified information. While prior research has focused on credibility judgments, less is known about how emotional responses shape engagement with such content. This study examines how emotional responses influence adolescents’ engagement with misinformation in a simulated social media context. A within-subject experiment was conducted with 44 Romanian high-school students (ages 16–19), who evaluated 18 war-related headlines presented in a feed-like format. Participants rated credibility, emotional responses, and their willingness to share and comment. Data were analyzed using mixed-effects models. Participants demonstrated baseline sensitivity to veracity, rating real headlines as more credible than false ones. However, engagement was more strongly associated with emotional responses than with perceived accuracy. High-arousal negative emotions—particularly fear and anger—predicted increased intentions to share and comment, whereas neutral responses were associated with minimal engagement. These findings show that adolescents’ online behavior reflects a dissociation between credibility judgments and engagement, with emotional responses playing a catalysing role. The study contributes to misinformation research by integrating cognitive and affective processes within a single experimental framework and highlights the importance of addressing not only cognitive but also emotional responses in media literacy interventions.

## Introduction

Social media has become a primary gateway to information for adolescents, shaping how they encounter, interpret, and respond to news. In the United States, recent nationally representative surveys indicate that about nine in ten teenagers use YouTube, with majorities also active on TikTok, Snapchat, and Instagram, and a substantial proportion reporting that they are online “almost constantly” (Pew Research Center, 2025). Similar patterns are observed in the United Kingdom, where internet access among young people is nearly universal, and older children and adolescents increasingly encounter news through digital platforms (Ofcom, 2024). Within these environments, entertainment, commentary, and news content appear side by side, often in headline-first formats and with limited source cues. This places adolescents in a position where they must make frequent credibility judgments under conditions that are fast-paced and cognitively demanding (Wineburg and McGrew, 2019).

These challenges become particularly salient in the context of the Russia–Ukraine war, where emotionally charged narratives circulate alongside verified reporting and misleading claims. A growing body of research shows that emotional content—especially content that conveys moral or threat-related signals—tends to spread more widely in online environments (Brady et al., 2017), particularly when reposted by concerned users themselves. At the same time, studies have demonstrated that relatively simple interventions, such as prompting users to consider accuracy, can reduce the likelihood of sharing misinformation without decreasing engagement with true information (Pennycook et al., 2021; Pennycook and Rand, 2022). Other approaches, including prebunking and lateral reading, similarly suggest that adolescents’ content evaluation strategies can be improved with targeted support (Roozenbeek et al., 2022; Wineburg and McGrew, 2019). Still, adolescent reactions or resilience against war-related online propagandas should be studied closer.

Importantly, existing research also indicates that adolescents are not uniformly susceptible to misinformation. Across large samples, participants tend to rate true headlines as more credible than false ones, suggesting a baseline sensitivity to veracity even in fast-paced, affectively loaded environments (Pennycook and Rand, 2019). Experimental findings further show that brief moments of reflection can selectively reduce belief in false information, pointing to the role of attention and cognitive control in shaping accuracy judgments (Bago et al., 2020). However, much of this work has focused on credibility judgments in isolation. Less is known about how these evaluations relate to emotional responses and, in turn, to the ways adolescents choose to engage with emotionally stimulating content.

This question is particularly relevant in Eastern European contexts, where proximity to the Russia–Ukraine conflict increases both exposure to and the emotional salience of war-related information. Romanian adolescents navigate a highly digital media environment in which social platforms function as important sources of news and commentary (CJI (Centrul pentru Jurnalism Independent) & PONT Group, 2025; Reuters Institute for the Study of Journalism, 2024, 2025). At the same time, the presence of institutional and independent fact-checking initiatives creates an information landscape in which accurate reporting and misleading claims coexist. Taken together, these conditions make Romania a useful naturalistic context for examining how adolescents evaluate and engage with conflict-related content in platform-like environments.

The present study builds on this literature by examining how adolescents evaluate the credibility of and respond emotionally to war-related headlines presented in a feed-like format. Rather than focusing solely on accuracy judgments, we consider how emotional responses relate to engagement behaviors, including the willingness to share and comment on content. Using a within-subject experimental design, the study captures how these processes unfold across both real and false headlines. and if the engagement with misinformation can me meaningfully managed through media literacy interventions targeting both cognitive and emotional control.

## Theoretical background

### Adolescents, social media, and news

Social media platforms have become a central component of adolescents’ everyday information environments. Similar patterns emerge across Europe, where studies document near-universal connectivity and early adoption of social networking and video platforms, with adolescents navigating mixed digital spaces in which communication, entertainment, and informational content coexist (Smahel et al., 2020). Reports from the Reuters Digital News Project further suggest that adolescents encounter news both intentionally and incidentally, often through visually oriented platforms such as TikTok, Instagram, and YouTube (Reuters Institute for the Study of Journalism, 2023, 2025).

Within these environments, news and public-affairs content are embedded in streams designed to maximize engagement. This is particularly evident during high-salience events such as the Russia–Ukraine war, where content may originate from a wide range of sources—including professional media, institutional actors, propaganda agencies, influencers, and peers—and is frequently presented in short, attention-grabbing formats. As a result, adolescents are required not only to evaluate the credibility of information but also to respond to it within fast-paced and emotionally charged contexts. This suggests that information processing in social media environments involves an ongoing interaction between cognitive evaluation and behavioral responses shaped by both heuristics and affective cues.

Research on online credibility indicates that, in headline-first or clip-first environments, users often rely on heuristics—such as familiarity, social endorsement, or interface cues—rather than engaging in systematic source evaluation (Metzger and Flanagin, 2013; Sundar, 2008; Hargittai et al., 2010; Messing and Westwood, 2014). These tendencies may be particularly pronounced among younger users, whose evaluative strategies are still developing. At the same time, regulatory reports suggest that adolescents may overlook or misinterpret platform signals such as sponsorship tags or content labels, further complicating credibility judgments in everyday browsing (Ofcom, 2024, 2025).

Importantly, this does not imply that adolescents are uniformly vulnerable. Research shows that many develop practical strategies for navigating digital environments and can improve their evaluative skills when provided with targeted support. Taken together, the literature points to a central tension: adolescents are capable of making credibility judgments, yet these judgments occur within environments that prioritize engagement, speed, and emotional salience. This raises an important question—how do these evaluative processes relate to the ways adolescents actually engage with content in such contexts?

### Why misinformation is compelling online

The appeal of misinformation in online environments can be understood as the result of interacting cognitive, affective, and social processes that shape how individuals evaluate and engage with content. Rather than reflecting a single deficit in reasoning, susceptibility to misinformation emerges from the way these processes operate under the conditions of platform-based information consumption.

At the cognitive level, perceived credibility is influenced by factors such as processing fluency and repetition. The illusory truth effect shows that repeated exposure to a claim increases its perceived accuracy, even when individuals are aware that it may be false (Fazio et al., 2015; Fazio, 2020). This effect extends to news headlines and is particularly pronounced in environments characterized by rapid and repeated exposure. Processing fluency—how easily information is recognized, read, or retrieved—can serve as a heuristic for truth, especially under time pressure. When such cues dominate, individuals are more likely to rely on intuitive judgments, e.g., based on formal resemblances to journalistic texts, whereas directing attention toward accuracy can encourage more deliberate evaluation (Brashier and Marsh, 2020; Brashier et al., 2020). Consistent with this account, even minimal interventions that prompt users to consider accuracy can reduce the influence of repetition on perceived truth (Brashier et al., 2020; Pennycook and Rand, 2021, 2022).

At the same time, cognitive evaluations of accuracy do not fully determine online behavior. Engagement decisions—such as whether to share content—are also shaped by social and motivational considerations. Individuals tend to share information that aligns with their identities, beliefs, or group norms, even when credibility judgments are uncertain or ambivalent (Papacharissi, 2015). In this sense, sharing is not only an informational act but also a form of social signaling, allowing individuals to express affiliation, reinforce group narratives, or participate in ongoing conversations.

These processes unfold within environments that are inherently affective. Emotional responses play a central role in shaping attention, memory, and behavior, particularly in social media contexts where high-arousal content is more likely to be prioritized and amplified. Content that combines moral language with emotional cues tends to spread more widely, with each additional moral-emotional element associated with increased diffusion (Brady et al., 2017). For adolescents navigating fast-paced and socially visible environments, this means that emotionally salient content is not only more likely to be encountered, but also more likely to prompt engagement.

Taken together, these mechanisms suggest that misinformation is compelling not only because it may appear credible, but because it aligns with how platform environments structure attention and interaction—through fluency, emotional salience, and social reinforcement. Importantly, credibility judgments and engagement behaviors do not necessarily operate in tandem. Individuals may recognize that content is questionable while still choosing to engage with it. This distinction underscores the need to examine credibility judgments and engagement behaviors jointly, rather than treating them as equivalent outcomes, particularly in high-salience contexts where emotional intensity and repeated exposure may amplify their effects.

### Analytic thinking and truth–falsehood discrimination

A longstanding assumption in education and cognitive science is that analytic thinking supports more accurate and discriminating judgments. In digital environments, this perspective has been extended to examine how individual differences in analytic reasoning relate to susceptibility to misinformation. Studies using cognitive reflection measures consistently show that individuals with higher levels of analytic thinking are less likely to believe false news, even when it aligns with their prior views, suggesting that errors in judgment often reflect insufficient engagement of analytic processing rather than purely motivated bias (Pennycook and Rand, 2019). Experimental findings further indicate that encouraging even brief deliberation can improve accuracy, particularly by reducing belief in false content (Bago et al., 2020). This pattern is supported by large-scale evidence, including meta-analytic work demonstrating a robust association between analytic thinking and the ability to distinguish between true and false information across contexts (Sultan et al., 2024).

At the same time, the role of related constructs such as Need for Cognition (Cacioppo and Petty, 1982) appears more complex. Some studies suggest that, once general cognitive ability is taken into account, NFC shows limited or inconsistent associations with susceptibility to misinformation (Hutmacher et al., 2024). Nonetheless, broader syntheses continue to link analytic engagement more generally to improved discernment in online environments (Pennycook and Rand, 2021), pointing to the importance of how individuals allocate attention and cognitive effort when evaluating information.

Alongside individual differences, a growing body of research highlights the effectiveness of minimal interventions that target analytic processing. Accuracy prompts—brief reminders to consider whether content is true—have been shown to reduce willingness to share misinformation without diminishing engagement with accurate content (Pennycook and Rand, 2021, 2022). Similarly, inoculation approaches that expose individuals to weakened examples of common manipulation techniques can build resistance that generalizes across topics and platforms (Lewandowsky and Van der Linden, 2021; Roozenbeek et al., 2022; Roozenbeek and Van der Linden, 2019). Instruction in lateral reading further improves adolescents’ ability to evaluate online sources, bringing their strategies closer to those used by expert fact-checkers (Wineburg and McGrew, 2017/2018; Brodsky et al., 2021).

Taken together, this literature shows that adolescents are cognitively capable of distinguishing between true and false information, and that, unlike smaller children, both individual differences and simple interventions can meaningfully improve their accuracy judgments. At the same time, these findings raise an important question: to what extent do improvements in accuracy translate into changes in engagement behavior in emotionally and socially dynamic online environments?

### Emotions, social incentives, and self-censorship

Emotions are an important part of how individuals process and respond to information. Beyond influencing how content is evaluated, emotional reactions shape attention and engagement with content (Papacharissi, 2015). In online environments, where content competes for attention and engagement is often immediate, emotionally arousing information tends to stand out more than neutral content and is more likely to be shared (Brady et al., 2017). Emotional arousal refers to the intensity of an emotional response, regardless of whether the emotion is positive or negative. Emotions such as fear and anger are particularly relevant in this context because, on the one hand, they have been associated with stronger engagement and a greater likelihood of interacting with online content (Rathje et al., 2021), and on the other, they are more likely to be generated by war-related content. At the same time, emotional responses are not limited to fear and anger. Emotional reactions vary in both valence and arousal (Scherer, 2005), and exposure to conflict-related content may elicit a broader range of emotions, including sadness, surprise, disgust, distrust, or even positive reactions. While different emotions may emerge in response to war-related information, previous research suggests that high-arousal emotions are more likely to be associated with active forms of online engagement. Accordingly, the present study explores a broader emotional spectrum and examines whether emotions characterized by higher levels of arousal are associated with stronger engagement behaviors.

Emotion is not merely a byproduct of online content; it plays a central role in shaping engagement. Large-scale analyses show that moral-emotional language significantly increases the likelihood of sharing, with these effects often amplified within ideologically aligned networks (Brady et al., 2017). Platform feedback mechanisms further reinforce this dynamic, as users learn over time that emotionally intense content—particularly content expressing outrage—tends to generate higher levels of engagement (Brady et al., 2021). In addition, individuals frequently overestimate the emotional intensity of others, contributing to the normalization of high-arousal responses and facilitating the spread of emotionally charged content (Goldenberg et al., 2020). More broadly, negative high-arousal emotions, such as anger, have been consistently linked to increased engagement and diffusion in social media environments (Rathje et al., 2021), with processes of emotional contagion further amplifying these effects (Kramer et al., 2014).

These affective dynamics are closely intertwined with social and reputational considerations. Although individuals may anticipate potential costs associated with sharing inaccurate information, such concerns do not operate uniformly across contexts. In socially aligned networks, emotional expression and identity signaling can be rewarded, even when the accuracy of content is uncertain, creating conditions in which affective and social incentives outweigh accuracy considerations (Rathje et al., 2021). At the same time, self-censorship dynamics suggest that individuals may withhold dissenting views in environments perceived as politically hostile, further shaping patterns of visible engagement (Matthes et al., 2018) which may be measured with a “willingness to self-censor” instrument.

Taken together, these findings indicate that engagement decisions in online environments are not determined by accuracy judgments alone. Instead, they reflect a dynamic interplay of emotional, social, and cognitive factors. This perspective helps explain why individuals may engage with content that they recognize as questionable, particularly in contexts where emotional intensity and social reinforcement are salient. However, from the perspective of limiting the spread of war-related propaganda, it is of importance to test ways to effectively contain emotionally engaging misinformation.

### Platformed war, narratives, and emotionalization in the Russia–Ukraine conflict

The Russia–Ukraine conflict unfolds within a media ecosystem shaped by platform logics, where information is rapidly produced, circulated, and interpreted through digital formats. On platforms such as TikTok, the war is often represented through short-form, creator-driven content that blends frontline footage, personal narratives, and memetic elements, rendering complex geopolitical events in accessible and emotionally engaging ways (Marino, 2024). At the same time, platforms such as Telegram function as central hubs for both information and propaganda, enabling the rapid circulation of official updates, community alerts, and unverified claims within large-scale networks (Bawa et al., 2025). Sometimes the same influencer channels host both reliable and misleading information, complicating credibility judgments and blurring the boundaries between reporting and manipulation. Broader system-level assessments further point to ongoing vulnerabilities to coordinated amplification and strategic influence (NATO Strategic Communications Centre of Excellence, 2023).

Within this ecosystem, conflict-related narratives are often constructed using emotionally salient frames, including threat, urgency, and moral evaluation. Linguistic analyses show that war news headlines tend to rely on condensed ambiguous structures, salience of antagonistic social actors, evaluative language, and strategic framing to capture attention and intensify emotional responses (Molek-Kozakowska, 2026; Molek-Kozakowska and Dragomir, 2026). These features align with broader platform dynamics that privilege high-arousal content signaling and tagging, helping to explain why emotionally charged war-related information is more likely to be noticed, shared, and discussed.

### The present study

Taken together, existing research points to a clear gap. While credibility judgments, emotional responses, and engagement behaviors have each been examined in prior work, they are typically studied in isolation. As a result, less is known about how adolescents evaluate (mis)information and respond to it emotionally at the same time, and how these processes jointly shape engagement in platform-like environments or how they can be better managed through interventions.

The present study addresses this gap by examining how adolescents evaluate and engage with war-related headlines presented in a feed-like setting. In particular, we focus on the role of emotional responses in shaping engagement behaviors, such as the willingness to share and comment on content. Using a within-subject experimental design, the study captures how credibility judgments and affective reactions unfold in response to both real and false headlines.

Specifically, we address three questions. First, do adolescents show baseline sensitivity to veracity, rating real headlines as more credible than false ones? Second, how do emotional responses to headlines (e.g., fear, anger, sadness, or neutrality) relate to willingness to share and comment on content? Third, does brief exposure to misinformation-related concepts correspond to directional changes in credibility judgments, particularly for false headlines?

By examining these processes together, the study aims to provide a more integrated account of how adolescents interact with information in social media environments, where evaluation and engagement often occur simultaneously and under the influence of emotional cues.

## Methods

### Participants and recruitment

Participants were 44 high-school students from Sibiu, Romania (grades 11–12; ages ≈16–19). They were recruited during a science outreach event organized by the university. All took part voluntarily and provided informed and/or parental consent. Procedures were approved by the Lucian Blaga University Ethics Committee (Approval No. 5303, 13.10.2025). It was ensured that the participants represented a fairly coherent cohort regarding their socio-economic status, cultural and linguistic background, media access, technological literacy and educational experience, considering the increased attention to preparedness in Romanian secondary education since the full scale war in Ukraine started.

### Design and materials

True headlines were sampled from the CORECON corpus and selected to represent common themes in Romanian coverage of the Russia–Ukraine conflict, including humanitarian support, military assistance, and security developments. Examples included “*Ucraina va primi cele şase tunuri Caesar suplimentare promise de Macron*” (“Ukraine will receive the six additional Caesar howitzers promised by Macron”) and “*Spania va trimite un mic număr de rachete Patriot în Ucraina*” (“Spain will send a small number of Patriot missiles to Ukraine”). False headlines were adapted from recurring misinformation narratives documented by Romania’s Ministry of Defence debunking initiative (Inforadar), including claims related to refugee privileges, military mobilization, and threats to Romanian security. Examples included “*Convoaie NATO intră în România în noaptea de 23 august; mobilizare secretă*” (“NATO convoys enter Romania on the night of August 23; secret mobilization”) and “*Primăria Siret plătește 2.000 € lunar fiecărui refugiat ucrainean*” (“The Siret City Hall pays €2,000 per month to every Ukrainian refugee”). To increase comparability across items, all headlines were presented in a standardized social-media-style format and were edited only minimally for consistency in presentation while preserving their original meaning and narrative structure.

To reflect characteristic features of conflict-related misinformation, fabricated headlines were constructed around recurrent discursive and framing patterns observed in Russia–Ukraine war coverage, including threat-oriented narratives, legitimizing and delegitimizing framings, and emotionally salient representations of conflict actors and events (Iliș, 2026). These items often conveyed high-stakes or imminent scenarios (e.g., military escalation or direct threats), designed to elicit stronger emotional responses. In contrast, real headlines were drawn from verified reporting and focused thematically on defensive preparedness, humanitarian support or administrative processes. As such, the stimulus set captures a contrast between high-arousal, threat-oriented content and lower-arousal informational content, while maintaining a realistic mix of narratives encountered in the Romanian information environment in 2025. At the same time, this design implies that emotional tone and veracity are not fully independent, while ensuring both ecological relevance and experimental control (Limonier, 2022).

To approximate everyday platform exposure, all stimuli were presented as short Romanian headlines in a feed-like, headline-first format. This design captures key features of social media browsing—such as rapid exposure, limited contextual cues, and emphasis on salience—while maintaining consistency across participants. In line with ethical considerations for adolescent samples, stimuli were restricted to text-based content and excluded graphic or highly sensational imagery.

Participants evaluated a total of 18 headlines within a within-subject design. In the pre phase (Q1–Q12), they rated 12 headlines—six verified as real (Q1–Q6) and six false (Q7–Q12). In the post phase (Q13–Q18), they evaluated six additional headlines (three real, three false). This structure enabled the examination of within-participant patterns in credibility judgments and engagement responses across multiple exposures.

For each headline, participants provided ratings on six measures using 1–5 Likert scales: perceived credibility, likelihood of sharing, likelihood of verifying via a second source, confidence in their judgment, and likelihood of commenting. Emotional responses were reported categorically and analyzed descriptively. Together, these measures capture both evaluative (credibility, confidence) and behavioral (sharing, commenting, verification) dimensions of engagement in a platform-like context.

Individual-difference measures were collected prior to the headline task. News literacy was assessed using 14 Likert-type items adapted from existing measures of news media literacy (e.g., Gudekli et al., 2022; Ashley et al., 2013). Need for Cognition was measured using a short version of the Need for Cognition scale (Cacioppo and Petty, 1982). Willingness to self-censor was assessed using an 8-item version of the Willingness to Self-Censor scale (Hayes et al., 2005), adapted for the context of online information sharing. Additional indicators included self-reported screen time, time spent consuming news, and typical platform use (categorical, multiple selections allowed).

Overall, the study used a controlled, feed-like experimental design implemented in a naturalistic educational setting. The study was conceived as an exploratory investigation of how credibility judgments, emotional responses, and engagement intentions relate to one another in a social media-like environment. Although participants were recruited based on school cohort membership and were not randomly assigned to conditions, the use of standardized stimuli and within-subject comparisons made it possible to examine consistent patterns in how adolescents evaluated and engaged with information.

### Procedure

Participants first completed the individual-difference measures, followed by headline-level evaluations in the pre phase (12 items). They then took part in a one-hour instructional and interactive session focused on misinformation, verification strategies, and algorithmic curation. The session in Romanian was delivered by two members of the research team: one with expertise in media, communication, and misinformation, who led the instructional component, and the other with a background in psychology, who administered the experimental procedure. This segment had two components. The first consisted of a short instructional module outlining how misinformation can exploit cognitive shortcuts (e.g., emotional arousal, motivated reasoning), paired with a compact verification routine adapted from the “Four Moves & a Habit” framework (i.e., checking existing knowledge, consulting original sources, reading laterally, and restarting the search process when needed). The second component involved an interactive task designed to make algorithmic curation visible in practice. Participants first engaged with interest-based content to generate a personalized, single-topic feed, and then compared it to a more diversified stream. This contrast made salient how everyday engagement patterns can shape—and potentially narrow—the range of information encountered online, creating conditions akin to “echo chambers” in which similar types of content are repeatedly reinforced.

Following this exposure, participants completed the post phase, evaluating six additional headlines using the same measures as in the pre phase.

### Data preparation

All analyses were conducted in R. Item responses on Likert scales were treated as numeric (1–5), with invalid entries coded as missing. For multi-item constructs—news literacy (NL1–NL14), need for cognition (NFC1–NFC6), and willingness to self-censor (WTSC1–WTSC8)—participant-level composite scores were computed as the mean of the corresponding items (higher values indicating higher levels of the construct). Continuous covariates included in the models were *z*-scored prior to analysis, while platform was treated as a categorical factor.

For the headline task, data were reshaped into long format. Phase was coded as pre (Q1–Q12) and post (Q13–Q18), and veracity as real (Q1–Q6, Q13–Q15) or fake (Q7–Q12, Q16–Q18). The primary outcome variable was perceived credibility (1–5). Additional behavioral measures included willingness to share (1–5), likelihood of commenting (binary), and verification and confidence ratings.

For analyses involving emotional responses, reported emotion labels (originally in Romanian) were grouped into nine categories: neutral (reference), fear, anger, sadness, surprise, disgust, contempt, distrust, and happiness, with infrequent responses retained under an “other” category.

### Sample characteristics

The analytic sample comprised 44 high-school students. Gender distribution was slightly female-majority: 23 identified as female (52.3%), 18 as male (40.9%), and 3 preferred not to say (6.8%). Self-reported daily screen time clustered in the middle ranges: 18 students (40.9%) reported 2–4 h/day, 16 (36.4%) 4–6 h/day, 6 (13.6%) > 6 h/day, and 4 (9.1%) < 1 h/day. Using conventional category midpoints (<1 h = 0.5; 2–4 h = 3; 4–6 h = 5; >6 h = 7), the implied average screen exposure was approximately 4 h/day. Thus, more than three-quarters of the cohort (77.3%) reported 2–6 h of daily use, consistent with contemporary adolescent media habits and providing ecological validity for a feed-based task that assesses credibility and engagement with headlines.

### Statistical analysis

We estimated linear mixed-effects models using the lmerTest package in R, including random intercepts for participant and item (question). Phase (pre vs. post) and veracity (real vs. fake) were included as fixed effects, with phase = pre and veracity = fake as reference levels.

The primary model tested the interaction between phase and veracity. Individual-difference measures were included as covariates rather than focal predictors. Given the exploratory nature of the study and the modest sample size, the aim was not to examine complex moderation effects, but rather to assess whether the observed patterns remained evident after accounting for theoretically relevant differences in news literacy, need for cognition, self-censorship, and media use. A second model extended this specification by including individual-difference and media-use covariates (z-scored prior to analysis), namely news literacy, need for cognition, willingness to self-censor, screen time, news consumption, and platform.

Degrees of freedom were estimated using Satterthwaite or Kenward–Roger approximations as implemented in R. To aid interpretation, we computed estimated marginal means (EMMs) which represent model-based adjusted group means and the difference-in-differences (DiD) contrast for the phase × veracity interaction using the emmeans package. The DiD contrast quantifies whether the change from the pre phase to the post phase differs between real and false headlines. Statistical significance was evaluated at *α* = 0.05 (two-tailed).

To examine engagement behaviors, we estimated separate mixed-effects models for (a) willingness to share (1–5 Likert scale, treated as approximately continuous) and (b) likelihood of commenting (binary), including random intercepts for participant and item. Emotion was entered as the sole fixed effect, with neutral as the reference category. Emotion labels were harmonized into nine categories (neutral, fear, anger, sadness, surprise, disgust, contempt, distrust, happiness), with infrequent responses grouped as “other.”

For the sharing model, we report estimated marginal means and pairwise contrasts relative to the neutral category with false discovery rate (FDR) adjustment. As a robustness check, we also fitted an ordinal cumulative-logit mixed model, which yielded substantively similar results. For commenting, we report odds ratios and predicted probabilities based on EMMs on the response scale.

Missing data were handled using maximum likelihood estimation. All statistical tests were two-tailed with *α* = 0.05.

## Results

We analyzed credibility ratings (1–5 scale) from 44 participants across 18 headlines using linear mixed-effects models with random intercepts for participant and item.

### Model 1: credibility judgments

Participants demonstrated baseline sensitivity to veracity. At pre-test, real headlines were rated as more credible than false ones, *b* = 0.63, SE = 0.27, *t*(≈18) = 2.33, *p* = 0.032 (Table 1).

**Table 1:** Credibility ratings by phase and veracity (Model 1).

Phase	Veracity	EMM	SE	95% CI
Pre	Fake	2.64	0.22	[2.18, 3.10]
Post	Fake	1.98	0.31	[1.34, 2.61]
Pre	Real	3.27	0.22	[2.81, 3.72]
Post	Real	3.23	0.31	[2.59, 3.87]

Credibility ratings for false headlines showed a directional decrease from pre- to post-test, *b* = −0.66, SE = 0.33, *t*(≈18) = −2.01, *p* = 0.060. However, the phase × veracity interaction was not statistically significant, *b* = 0.63, SE = 0.47, *t*(≈18) = 1.35, *p* = 0.193, indicating that the increase in the real–fake credibility gap over time did not reach significance.

Descriptively, the real–fake credibility gap increased from 0.64 at pre-test to 1.25 at post-test. Consistent with this pattern, the difference-in-differences (DiD) contrast suggested a positive but non-significant improvement in discernment, *b* = 0.63, SE = 0.52, 95% CI [−0.46, 1.71], *t* = 1.20, *p* = 0.243 (Table 2).

**Table 2:** Difference-in-differences contrast for credibility ratings (Model 1).

Contrast	*b*	SE	95% CI	*df*	*t*	*p*
DiD (post − pre, real vs. fake)	0.63	0.52	[−0.46, 1.71]	22.52	1.20	0.243

### Model 2: credibility judgments with covariates

Including individual-difference and media-use covariates did not alter the pattern of results. The effect of veracity remained, while the phase × veracity interaction continued to be non-significant. None of the covariates reached statistical significance.

### Emotion–engagement

Beyond credibility judgments, emotional responses were strongly associated with engagement behaviors. For sharing, neutral responses were linked to the lowest levels of intended engagement, whereas high-arousal negative emotions were associated with higher sharing intentions. In particular, fear [M ≈ 2.26, 95% CI (1.96, 2.57)] and anger [M ≈ 2.17, 95% CI (1.84, 2.50)] showed the highest levels, with sadness and surprise occupying an intermediate range.

Pairwise contrasts confirmed that both fear and anger were associated with significantly higher sharing intentions compared to neutral, with fear showing the largest effect (*b* = 0.87, *p* < 0.001; Table 3). Sadness and surprise also differed significantly from neutral, although with smaller effect sizes, while other emotions showed weaker or non-significant differences.

**Table 3:** Pairwise contrasts for emotion effects on sharing intentions.

Contrast	*b*	SE	95% CI	*df*	*t*	*p*
Fear vs. neutral	0.87	0.12	[0.53,1.2]	547.37	7.16	< 0.001
Sadness vs. neutral	0.45	0.14	[0.07, 0.82]	258.81	3.28	0.003
Anger vs. neutral	0.77	0.13	[0.39, 1.14]	505.12	5.70	< 0.001
Surprise vs. neutral	0.45	0.09	[0.22, 0.69]	742.95	5.31	< 0.001
Disgust vs. neutral	0.54	0.27	[−0.2, 1.28]	757.93	2.01	0.067
Contempt vs. neutral	0.35	0.22	[−0.26, 0.97]	771.35	1.60	0.143
Distrust vs. neutral	−0.07	0.81	[−2.33, 2.19]	751.47	−0.09	0.931
Happiness vs. neutral	0.48	0.15	[0.06, 0.89]	764.83	3.19	0.003
Other vs. neutral	0.22	0.25	[−0.46, 0.9]	756.03	0.89	0.423

A similar pattern emerged for commenting behavior. Predicted probabilities indicated that anger was associated with the highest likelihood of commenting (≈ 18–19%), followed by distrust and fear, whereas neutral responses were associated with near-zero probabilities. Other emotions were linked to substantially lower engagement levels.

Overall, emotionally charged headlines—particularly those eliciting anger and fear—were substantially more likely to prompt active engagement, both in terms of sharing and commenting. Odds ratios from the logistic model (Table 4) confirmed this pattern, although some estimates (e.g., distrust) were imprecise due to sparse observations and should be interpreted with caution.

**Table 4:** Odds ratios for emotion predicting commenting behavior (mixed-effects logistic model).

Predictor	OR	95% CI	SE	*z*	*p*
Intercept	0.001	[0.0001, 0.009]	0.001	−6.29	< 0.001
Fear	34.05	[6.26, 185.12]	29.41	4.08	< 0.001
Sadness	8.43	[1.36, 52.33]	7.85	2.29	0.022
Anger	217.53	[40.14, 1178.89]	187.57	6.24	< 0.001
Surprise	5.85	[1.18, 29.10]	4.79	2.16	0.031
Disgust	116.85	[9.90, 1379.18]	147.16	3.78	< 0.001
Contempt	30.41	[1.68, 549.83]	44.92	2.31	0.021
Distrust	5.72	[0.00, —]	111.11	0.09	0.928
Happiness	18.22	[3.09, 107.54]	16.50	3.20	0.001
Other	0.01	[0.00, —]	0.93	−0.05	0.958

## Discussion

The present study examined how adolescents evaluate and engage with war-related information in a social media context, with a particular focus on the role of emotional responses. Two main patterns emerged. First, adolescents demonstrated baseline sensitivity to veracity, consistently rating real headlines as more credible than false ones. Second, and more centrally, emotional responses—particularly high-arousal negative emotions such as fear and anger—were strongly associated with increased willingness to share and comment on content. Taken together, these findings suggest that adolescents’ engagement with information in platform environments is shaped not only by what they believe, but also—perhaps more strongly—by how content makes them feel.

The presence of baseline truth discernment aligns with prior research showing that individuals, including younger users, are not uniformly susceptible to misinformation and can differentiate between true and false content under typical conditions (Pennycook and Rand, 2019). At the same time, the observed directional decrease in credibility for false headlines following exposure to misinformation-related concepts is consistent with work demonstrating that relatively small shifts in attention or reasoning can improve the rejection of false information (Bago et al., 2020; Pennycook et al., 2021). Rather than indicating a strong intervention effect, this pattern suggests that evaluative processes are responsive to contextual cues even within short, ecologically realistic exposures.

Crucially, credibility judgments did not fully determine engagement behavior. Emotional responses were strongly associated with how adolescents reported they would act on content. War-related headlines that elicited fear and anger were associated with higher intentions to share and comment, whereas neutral responses were linked to minimal engagement. This pattern is consistent with earlier research demonstrating that high-arousal affect increases content diffusion in online environments (Brady et al., 2017; Berger and Milkman, 2012; Stieglitz and Dang-Xuan, 2013) but it needs constant monitoring as regards media users that might become desensitized to ubiquitous emotionality or have higher levels of preparedness regarding conflict-mongering at a time of war.

What we find particularly interesting is that credibility judgments and engagement intentions did not appear to move together. On the one hand, participants generally distinguished between real and false headlines, suggesting a relatively high degree of sensitivity to veracity. On the other hand, intentions to share and comment were more closely linked to emotional responses than to perceived accuracy. This suggests that evaluating information and deciding whether to engage with it may be partly distinct processes as hypothesized by Papacharissi (2015) over a decade ago. In other words, recognizing that a piece of content is questionable does not necessarily prevent engagement with it if it evokes a strong emotional reaction. While this pattern should be interpreted cautiously, it points to the importance of redefining the notions of individual and collective misinformation engagement in terms of credibility and towards other predictors of engagement, such as emotional responses.

The above distinction may also have implications for how misinformation engagement is conceptualized. Much of the existing literature has focused on improving the accuracy of credibility judgments, often assuming that better discernment will translate into more selective engagement with content. The present findings suggest that for adolescents in particular, engagement decisions may be shaped not only by what they believe about a piece of information, but also by how they feel about it. From this perspective, planning effective misinformation engagement interventions requires including the skillful management of evaluative and affective processes, through for example pausing, as indicated by Fazio (2020), rather than treating engagement as a straightforward consequence of credibility misjudging remedied with more fact-checking.

From a process perspective, these results point to a partial dissociation between evaluation and action. While adolescents demonstrated the capacity to distinguish between real and false headlines, their willingness to engage with content was more strongly correlated with emotional responses than with perceived accuracy. With so many young people active online, this distinction helps explain why misinformation may continue to spread even in contexts where users possess a relatively sufficient degree of discernment, but find it hard to control their affect.

These findings also highlight the importance of considering the broader informational ecology of social media platforms. Content that is emotionally salient is more likely to be encountered, remembered, and shared, due both to algorithmic amplification and to social reinforcement mechanisms (Brady et al., 2017). In such environments, regulations and interventions that focus exclusively on improving accuracy judgments may have limited impact unless they also address the affective and motivational dynamics that drive engagement. Media literacy efforts may therefore benefit from incorporating strategies that increase awareness of linguistic and visual emotional cues, complementing existing approaches centered on claim verification and source evaluation, as has been common so far (Pennycook et al., 2021; Wineburg and McGrew, 2019).

The absence of significant effects for individual-difference and media-use variables should be interpreted with caution. These measures were included primarily as controls rather than as focal explanatory variables, and the study was not designed to examine more complex interaction or moderation effects. While prior research has identified modest associations between analytic thinking and susceptibility to misinformation (Bago et al., 2020; Pennycook and Rand, 2019), the present study was not optimized to detect such effects. The combination of a modest sample size and limited reliability for some measures may have attenuated these relationships.

Taken together, the study contributes to the literature by linking credibility judgments, emotional responses, and engagement behaviors that can be subjected to management within a single experimental framework. Rather than treating misinformation susceptibility as a purely cognitive deficit, the findings support a more integrated account in which adolescents’ online behavior reflects the interaction of evaluative and affective processes within platform-based environments.

## Limitations

Several limitations should be considered when interpreting these findings. The sample size was modest, which may have limited statistical power for detecting smaller effects, particularly interactions. Accordingly, the study should be viewed as exploratory in nature, with the primary goal of identifying patterns and generating evidence that can be tested in larger and more diverse samples. At the same time, the consistency of patterns across items and outcomes suggests that the observed effects reflect underlying tendencies rather than isolated results.

The sample was also relatively homogeneous, consisting of adolescents from a similar social, cultural and educational context. While this allowed for a more controlled examination of responses within a specific developmental environment, it also limits the generalizability of the findings. Accordingly, the study is best understood as providing focused, exploratory evidence rather than population-level estimates.

The post-test phase included fewer items than the pre-test phase, reflecting a deliberate effort to preserve attention and reduce fatigue in a classroom-based setting. Although this may have reduced the precision of some estimates, the overall pattern of results remained stable across phases.

In addition, emotional responses and engagement intentions were measured concurrently. As a result, the present design does not permit causal conclusions regarding the direction of these relationships. Accordingly, the findings support associations between emotional responses and engagement intentions, but do not establish that emotional responses cause adolescents to share or comment on content. Future research employing experimental manipulations of emotional content would be better positioned to examine the causal mechanisms linking emotional responses and engagement behavior.

An additional limitation concerns the relationship between veracity and emotional framing within the stimulus set. To preserve ecological validity, the fabricated headlines were designed to reflect common features of online misinformation, including urgency, threat framing, and emotionally salient narratives. Consequently, some characteristics associated with misinformation (e.g., threat framing and emotional salience) were not fully independent from headline veracity. While this approach captures the types of content adolescents may realistically encounter online, it limits the extent to which the present study can distinguish between the effects of misinformation status itself and the effects of specific framing characteristics. Future research could examine these factors separately by independently manipulating veracity, threat framing, and emotional salience.

Finally, the stimuli were restricted to non-graphic, text-based headlines within a single geopolitical context. This design ensured ethical appropriateness and experimental consistency, while capturing a relevant slice of the contemporary information environment. Future research should examine whether these findings generalize to other formats (e.g., visual or video content) and to different topical domains.

## Practical implications for schools and educators

The present findings suggest that effective media literacy efforts should address not only how adolescents evaluate information, but also how emotional responses shape their engagement with it. In platform environments where high-arousal content is more likely to be noticed, remembered, and shared, brief interventions that redirect attention and slow down reactive responses may be particularly impactful. For upper-secondary classrooms (Grades 11–12; approximately 16–18 years old), three principles can be implemented with minimal time investment.

### Make accuracy salient at the moment of engagement

Introduce short, repeatable routines that prompt students to pause and evaluate accuracy before reacting. A simple 60-s exercise—presenting a post and asking students to rate its likely accuracy (1–5) with a brief justification—can shift attention from emotional and social cues toward evaluative processing. Crucially, this pause should be framed not as avoidance of emotionally charged content, but as a way to interrupt automatic, affect-driven responses that often precede sharing or commenting. Extending the routine to include alternative response options (e.g., ignoring, verifying, correcting, or responding supportively) reinforces the idea that engagement is a choice shaped by both accuracy and social considerations.

### Teach fast, transferable verification strategies

Rather than emphasizing detailed source analysis, instruction should prioritize rapid, actionable routines that fit platform use. A minimal lateral-reading strategy—leaving the page, consulting external sources, and locating original reporting—can be modeled in minutes and practiced collaboratively. This approach aligns with how credibility judgments occur in real-world browsing and helps students externalize verification rather than relying on surface cues. Over time, repeated use can transform verification from a task into a, such as the posts’ mimicry of journalistic style habitual response.

### Build recognition of emotional and rhetorical cues

Given the strong link between high-arousal emotions and engagement, students benefit from learning to identify common manipulation techniques that exploit emotional reactions. Short prebunking activities can introduce patterns such as emotional loading, false dichotomies, scapegoating, or agentless authority. Asking students to label these tactics and rewrite content in neutral terms helps decouple emotional intensity from perceived importance and reduces the likelihood of reactive sharing.

These practices can be integrated into existing subjects (e.g., history, language, civics) and implemented incrementally. Light-touch assessments—such as brief justifications, exit ratings, or verification checklists—are sufficient to reinforce learning without increasing workload. Establishing classroom norms around pausing before sharing, checking sources, and acknowledging uncertainty can further support reflective engagement. Where possible, incorporating behavioral indicators (e.g., whether students open external sources) may provide a more direct link between instruction and online behavior.

Overall, these strategies target the key mechanisms identified in the present study: the tendency for emotional responses to drive engagement and the need for simple, scalable interventions that introduce friction into fast, platform-based decision-making. By combining accuracy-focused prompts, rapid verification routines, and awareness of emotional cues, educators can support more reflective and intentional interaction with digital information.

## Conclusion

The present study highlights a central pattern in adolescents’ interaction with war-related information in social media environments: while adolescents demonstrate an emerging ability to distinguish between real and false content, their engagement with that content is strongly shaped by emotional responses. Participants showed baseline sensitivity to veracity, alongside a directional improvement in the evaluation of false headlines following brief exposure to misinformation-related concepts. Although modest, this pattern is consistent with evidence that small shifts in attention can support more accurate judgments in fast, emotionally charged environments.

More centrally, the findings indicate that emotional responses were consistently associated with higher levels of intended engagement. High-arousal negative emotions—particularly fear and anger—were associated with greater willingness to share and comment, whereas neutral responses were linked to minimal engagement. This dissociation between credibility judgments and engagement behavior suggests that understanding information spread in digital environments requires integrating cognitive and affective processes, rather than treating them as equivalent.

From an applied perspective, the results point to the value of interventions that address not only accuracy, but also emotional responses to content. Brief, scalable practices—such as help manage the accuracy prompts, lateral reading routines, and recognition of emotional and rhetorical cues—may introduce self-monitoring and better control over into rapid, affect-driven engagement decisions and support more reflective interaction with online information.

Taken together, these findings suggest that improving adolescents’ ability to evaluate information, while important, may be insufficient on its own. In platform environments shaped by speed, repetition, and emotional intensity, how content makes users feel may be as important as whether they recognize it as true or false.

## Data Availability

The raw data supporting the conclusions of this article will be made available by the authors, without undue reservation.
