# Correction: Emotional responses and engagement with misinformation in adolescents

**DOI:** 10.3389/fpsyg.2026.1939054

**Published:** 2026-07-29

**Authors:** Ecaterina Ilis, Razvan Ilis, Katarzyna Molek-Kozakowska, Simina-Maria Terian

**Affiliations:** 1 Faculty of Letters and Arts, Lucian Blaga University of Sibiu, Sibiu, Romania; 2 Faculty of Social and Human Sciences, Lucian Blaga University of Sibiu, Sibiu, Romania; 3 Institute of Linguistics, University of Opole, Opole, Poland

**Keywords:** adolescents, credibility, emotion, misinformation, news literacy, Russia–Ukraine war, social media

Affiliation Faculty of Social and Human Sciences, Lucian Blaga University of Sibiu, Sibiu, Romania was omitted from the paper. This affiliation has now been added for author Razvan Ilis.

The original version of this article has been updated.

